# Inverse relationship between serum hsCRP concentration and hand grip strength in older adults: a nationwide population-based study

**DOI:** 10.18632/aging.101529

**Published:** 2018-08-16

**Authors:** Beom-Jun Kim, Seung Hun Lee, Mi Kyung Kwak, Carlos M Isales, Jung-Min Koh, Mark W Hamrick

**Affiliations:** 1 Division of Endocrinology and Metabolism, Asan Medical Center, University of Ulsan College of Medicine, Seoul 05505, South Korea; 2 Department of Cellular Biology and Anatomy, Medical College of Georgia, Augusta University, Augusta, GA 30912, USA; 3 Department of Orthopedic Surgery, Medical College of Georgia, Augusta University, Augusta, GA 30912, USA

**Keywords:** inflammation, hand grip strength, muscle strength, sarcopenia, high sensitivity C-reactive protein

## Abstract

Despite the potential detrimental effects of systemic inflammation on muscle mass, which is mainly observed in patients with pathologic diseases, its role in muscle strength, especially in a healthy general population reflecting subclinical low-grade inflammation, is unclear. This is a nationally representative population-based, cross-sectional study from the Korea National Health and Nutrition Examination Survey, which enrolled 1,036 men aged ≥50 years and 1,080 postmenopausal women. After adjustment for confounders, serum high-sensitivity C-reactive protein (hsCRP) level was inversely associated with hand grip strength (HGS) in men. Consistently, compared with men in the lowest serum hsCRP quartile, those in the highest quartile showed a significant lower HGS, with a linear decrease of HGS across increasing serum hsCRP quartiles. Men with low muscle strength had 74.2% higher serum hsCRP than those without, and each standard deviation increment in serum hsCRP was associated with a multivariate-adjusted odds ratio of 1.35 for the risk of low muscle strength in men. However, these associations were not statistically significant in women. These findings provide clinical evidence that chronic subclinical low-grade inflammation may contribute to the deterioration of muscle strength seen with aging, especially in men.

## Introduction

Hand grip strength (HGS) is an easily accessible and convenient indicator of overall muscle strength, with good test-retest reliability, validity, and responsiveness [1,2]. Low HGS has been linked to various comorbidities, such as hypertension, diabetes, cardiovascular disease, stroke, and chronic obstructive pulmonary disease, as well as the associated high mortality [3–7]. Given these links, the intriguing suggestion has been made that grip strength might act as a “biomarker of aging” across the life course [8,9].

Importantly, muscle strength is not dependent solely on muscle mass, and the relationship between muscle strength and mass is not linear [10–12]. Therefore, more consideration of muscle strength per se, besides muscle mass, in research and practice is necessary to fully understand the roles of potential effectors on muscle health.

Chronic low-grade inflammation, which is quite different from the acute inflammatory process induced by infection or tissue injury, is involved in the development and progression of age-related diseases, such as atherosclerosis, osteoporosis, diabetes, and dementia [13–15]. The sustained increase of oxidative stress-induced redox imbalance and the accumulation of pro-inflammatory mediators during aging could contribute to these adverse health outcomes [16,17]. Many lines of evidence from experimental studies now indicate that inflammation also has a direct detrimental effect on muscle metabolism [18,19]. For example, inflammatory cytokines can modulate signaling pathways involved in muscle homeostasis, leading to an imbalance between protein synthesis and proteolysis [20,21]. In view of these findings, several epidemiological studies have been performed to assess the role of high-sensitivity C-reactive protein (hsCRP), an indicator of systemic inflammation, as a risk marker for sarcopenia. However, most of these studies have focused only on muscle mass and been performed in patients with existing pathologic conditions, such as cancer, chronic obstructive pulmonary disease, end-stage renal disease, and ulcerative colitis [22–26]. Furthermore, clinical studies relating serum hsCRP level to muscle strength, especially in a healthy general population reflecting subclinical low-grade inflammation, are limited. With the aim to resolve these issues, the present study evaluated serum hsCRP concentration in relation to HGS in community-dwelling healthy older adults.

## RESULTS

Table 1 provides the clinical characteristics in each gender according to serum hsCRP concentration. In men, participants with higher hsCRP level (above median; ≥0.7 mg/L) were older and had higher weight, body mass index (BMI), serum total cholesterol, and systolic blood pressure (BP) than those with lower hsCRP level (below median; <0.7 mg/L), whereas there were no differences in height, smoking and drinking habits, resistance exercise, fasting plasma glucose, and diastolic BP between two groups. In women, participants with higher hsCRP level (above media; ≥0.7 mg/L) had higher weight, BMI, fasting plasma glucose, and systolic BP, and had lower height than those with lower hsCRP level (below median; <0.7 mg/L). However, there were no differences in age, smoking and drinking habits, resistance exercise, serum total cholesterol, and diastolic BP between two groups. In crude analyses, participants with higher hsCRP level had significantly lower HGS than those with lower hsCRP level in both men and women.

**Table 1:** Baseline characteristics of the study population according to serum hsCRP level.

Variables	Men (*n* = 1,036)				Women (*n* = 1,080)		
	hsCRP below median (<0.7 mg/L, *n* = 504)	hsCRP above median (≥0.7 mg/L, *n* = 532)	*P*		hsCRP below median (<0.7 mg/L, *n* = 555)	hsCRP above median (≥0.7 mg/L, *n* = 525)	*P*
Age (years)	**60.4 (59.5–61.2)**	**61.8 (60.8–62.8)**	**0.027**		61.9 (61.1–62.8)	62.8 (61.7–63.8)	0.236
Weight (kg)	**67.2 (66.1–68.3)**	**69.4 (68.5–70.3)**	**0.001**		**56.3 (55.6–57.0)**	**59.7 (58.7–60.8)**	**<0.001**
Height (cm)	167.9 (167.2–168.6)	167.5 (166.9–168.0)	0.326		**154.9 (154.4–155.5)**	**154.1 (153.5–154.7)**	**0.042**
Body mass index (kg/m^2^)	**23.8 (23.5–24.1)**	**24.7 (24.4–24.9)**	**<0.001**		**23.5 (23.2–23.8)**	**25.1 (24.8–25.5)**	**<0.001**
Smoking habit (%)			0.105				0.465
Never Past Current	18.3 56.5 25.2	14.5 53.4 32.1			93.0 4.1 2.9	91.4 4.0 4.6	
Drinking habit (%)			0.651				0.548
No	35.5	37.0			80.2	77.6	
Moderate	46.5	43.1			18.8	20.9	
Heavy	18.0	19.9			1.0	1.5	
Resistance exercise (%)			0.194				0.326
No	63.6	69.6			83.3	86.9	
Intermittent	15.7	13.6			9.3	7.4	
Regular	20.7	16.8			7.4	5.7	
Fasting plasma glucose (mg/dL)	105.7 (103.2–108.3)	109.5 (105.9–113.2)	0.086		**100.0 (98.0–102.0)**	**107.5 (104.6–110.5)**	**<0.001**
Serum total cholesterol (mg/dL)	**185.0 (181.3–188.6)**	**190.6 (187.3–194.0)**	**0.018**		201.1 (197.3–204.8)	201.4 (197.9–204.9)	0.891
Systolic BP (mmHg)	**123.0 (121.4–124.5)**	**126.7 (125.1–128.2)**	**0.001**		**122.7 (121.0–124.4)**	**125.2 (123.5–127.0)**	**0.046**
Diastolic BP (mmHg)	77.4 (76.4–78.4)	78.5 (77.6–79.5)	0.111		74.9 (73.9–75.9)	75.0 (74.0–76.0)	0.922
Hand grip strength (kg)	**39.8 (39.0–40.6)**	**38.5 (37.7–39.2)**	**0.013**		**24.5 (24.0–25.0)**	**23.6 (23.1–24.2)**	**0.022**

Values are presented as mean with 95% confidence intervals unless otherwise specified. *P* values were determined using the Student's *t*-test for continuous variables and the χ2 test for categorical variables. Bold numbers indicate statistically significant values. hsCRP, high sensitivity C-reactive protein.; BP, blood pressure.

Multiple linear regression analyses were performed to determine whether serum hsCRP level was independently associated with HGS (Table 2). Before and after adjustment for confounders including age, BMI, smoking and drinking habits, resistance exercise, fasting plasma glucose, serum total cholesterol, and systolic BP, serum hsCRP level was inversely associated with HGS in men. However, their relationship was not observed in women, regardless of adjustment models.

**Table 2:** Multiple linear regression analysis to determine whether serum hsCRP level is independently associated with hand grip strength.

Adjustment	Dependent variable: hand grip strength		
	β	SE	*P*
Men			
Unadjusted	**–0.342**	**0.115**	**0.003**
Age and BMI	**–0.262**	**0.091**	**0.005**
Multivariable	**–0.274**	**0.084**	**0.001**
Women			
Unadjusted	–0.177	0.091	0.052
Age and BMI	–0.102	0.094	0.280
Multivariable	–0.093	0.095	0.325

The Enter method is applied to this model with hand grip strength (kg) as a dependent variable, and with serum hsCRP level (mg/L) as an independent variable. Multivariable adjustment model includes age, body mass index, smoking and drinking habits, resistance exercise, fasting plasma glucose, serum total cholesterol, and systolic blood pressure as confounding factors. **Bold** numbers indicate statistically significant values. β, regression coefficient; SE, standard error; hsCRP, high sensitivity C-reactive protein.

**Figure 1:**
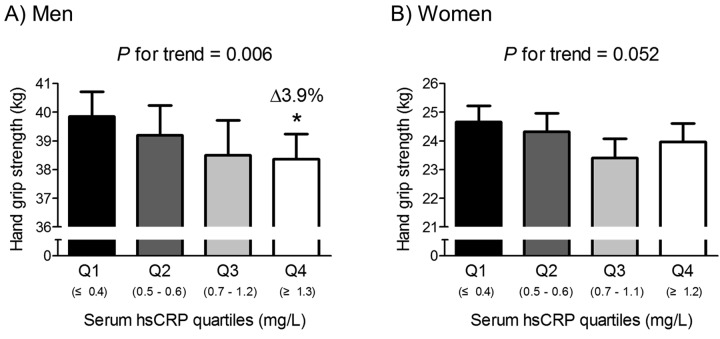
Hand grip strength according to serum hsCRP quartiles in men (**A**) and women (**B**). Delta (Δ) indicates a change in hand grip strength from the lowest quartile (Q1). Values are presented as the estimated mean with 95% confidence intervals after adjustment for confounding factors using analysis of covariance (ANCOVA). Confounding variables include age, body mass index, smoking and drinking habits, resistance exercise, fasting plasma glucose, serum total cholesterol, and systolic blood pressure. ^*^Statistically significantly different from the Q1 by ANCOVA. hsCRP, high sensitivity C-reactive protein.

To identify whether the association between serum hsCRP level and HGS is gradual or has a threshold effect, we divided the subjects into quartiles, according to serum CRP level in each gender (Fig. 1). In men, HGS linearly decreased across increasing serum hsCRP quartiles after adjustment for age, BMI, smoking and drinking habits, resistance exercise, fasting plasma glucose, serum total cholesterol, and systolic BP. Consistently, men in the highest serum hsCRP quartile (Q4) showed significantly lower HGS than those in the lowest quartile (Q1) (*P* = 0.011). Conversely, in women, there was no significant difference in HGS among serum hsCRP quartiles.

**Figure 2:**
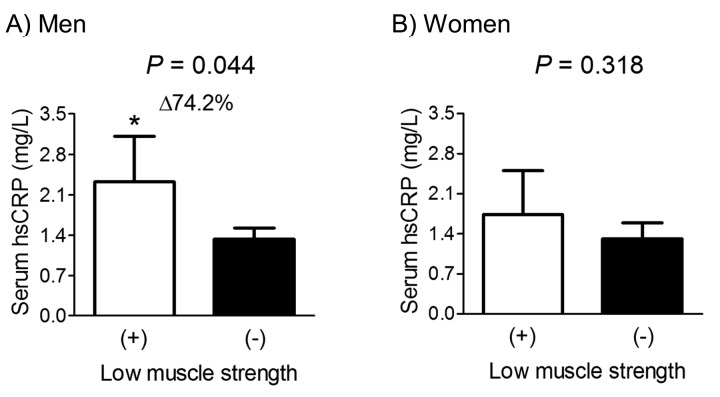
Difference in serum hsCRP concentration between participants with and without low muscle strength in men (**A**) and women (**B**) aged ≥65 years. Delta (Δ) indicates a change in serum hsCRP level from the control. Values are presented as the estimated mean with 95% confidence intervals after adjustment for confounding factors using analysis of covariance (ANCOVA). Confounding variables include age, body mass index, smoking and drinking habits, and resistance exercise, fasting plasma glucose, serum total cholesterol, and systolic blood pressure. ^*^Statistically significantly different from the control by ANCOVA. hsCRP, high sensitivity C-reactive protein.

Given the Korean-specific HGS cut-off values for low muscle strength in elderly populations aged ≥65 years are 28.6 (men) and 16.4 kg (women) [27], we performed the subgroup analyses in these subjects (Fig. 2). Among 460 men and 476 women aged ≥65 years, 16.1% male and 13.4% female subjects belonged to the low muscle strength group, based on HGS. After adjustment for confounders, subjects with low muscle strength had 74.2% higher serum hsCRP level than those without this condition, with statistical significance in men. However, serum hsCRP level was not significantly different between women with and without low muscle strength.

Multiple logistic regression analyses were undertaken to investigate the independent effect of serum hsCRP level on the low muscle strength (Table 3). After adjustment for age, BMI, smoking and drinking habits, resistance exercise, fasting plasma glucose, serum total cholesterol, and systolic BP, each standard deviation (SD) increment in serum hsCRP was associated with a multivariate-adjusted odds ratio (OR) of 1.35 for the risk of low muscle strength in men aged ≥65 years. However, the OR for low muscle strength, according to the serum hsCRP level, was not statistically significant in women, regardless of adjustment models.

**Table 3:** Multiple logistic regression analyses to determine the odds ratios for low muscle strength according to serum hsCRP level in participants aged ≥65 years.

Adjustment	Men			Women	
	^*^Odds ratio (95% CIs) for low muscle strength	*P*		^*^Odds ratio (95% CIs) for low muscle strength	*P*
					
Unadjusted	**1.28 (1.05–1.57)**	**0.017**		1.15 (0.93–1.42)	0.198
Age and BMI	**1.27 (1.01–1.61)**	**0.045**		1.16 (0.93–1.45)	0.182
Multivariable	**1.35 (1.04–1.76)**	**0.027**		1.12 (0.91–1.39)	0.279

Multivariable adjustment model includes age, body mass index, smoking and drinking habits, resistance exercise, fasting plasma glucose, serum total cholesterol, and systolic blood pressure as confounding factors. Bold numbers indicate statistically significant values. hsCRP, high sensitivity C-reactive protein; CI, confidence interval.

^*^Per standard deviation increment in serum hsCRP level (2.32 mg/L for men and 2.01 mg/L for women).

Finally, we not only adjusted for smoking and drinking habits, and resistance exercise in the multivariable analyses, but we also employed a stratified analyses according to these factors in men. As presented in Supplementary Table 1, there were consistent tendencies showing the inverse relationships between serum hsCRP level and HGS in all stratified subgroups, after adjustment for age, BMI, fasting plasma glucose, serum total cholesterol, and systolic BP in men. However, these associations were dominant with a statistical significance in past and current smoking, no drinking, and no and regular exercise subgroups.

## DISCUSSION

CRP, predominantly produced in the liver, is a member of the pentraxin family of innate immune recognition proteins and is regarded as a reliable marker of systemic inflammation [28]. The availability of sensitive immunoassays for hsCRP has enabled the measurement and comparison of very low CRP levels in the blood. In this nationally representative cohort of older adults, we observed that serum hsCRP level was inversely associated with HGS in men. Consistently, men with low muscle strength had markedly higher serum hsCRP level, and the risk for low muscle strength was 35% increased per SD increment in serum hsCRP level in men. However, these associations were not statistically significant in women. To the best of our knowledge, this is the first report showing the relationship between serum hsCRP level and HGS in a healthy Asian general population.

Several clinical studies have measured serum CRP levels in an attempt to elucidate the role of inflammation in the pathogenesis of sarcopenia. In general, as discussed in a recent meta-analysis article [29], sarcopenia seems to be associated with elevated serum CRP levels. However, the selected studies were highly heterogeneous and defined sarcopenia largely based on muscle mass [22–26]. Also, most studies were performed in patients with diseases affecting serum CRP level and/or muscle metabolism, in a hospital setting [22–26]. Therefore, although these studies have been devoted to understanding the possible involvement of systemic inflammation in human muscle health, the findings could not be generalized to non-pathological aging processes. In this context, our present study may have important implications in suggesting that chronic inflammation, even subclinical low-grade chronic inflammation, could be associated with low muscle strength in community-dwelling older adults.

Several possible mechanisms may link chronic low-grade inflammation and adverse muscle health during aging. First, accumulated data has suggested that pro-inflammatory cytokines, which are critical mediators of inflammatory processes, are upregulated due to an age-related redox imbalance [16], and then directly participate in modulating the molecular pathways related to protein synthesis and degradation [30]. For example, tumor necrosis factor-α is known to directly aggravate muscle catabolism by suppressing the Akt (also known as protein kinase B)/mTOR (mammalian target of rapamycin) pathway [18,20]. Second, increased expression of several important negative regulators, such as atrogin-1, muscle ring finger-1, nuclear factor-kappa B, and myostatin, has been proposed to enhance muscle wasting related to inflammation [31]. Third, pro-inflammatory cytokines may antagonize the anabolic effects of growth hormone and insulin-like growth factor 1, key hormones for the growth and differentiation of skeletal muscle, leading to negative muscle protein balance during aging [20,21]. Fourth, there is a possibility that circulating cytokines may induce contractile dysfunction of muscle proteins and thus cause diminished muscle force production, independently of protein loss [32].

A particularly interesting observation in the present study is that the inverse correlation of serum hsCRP level with HGS was more dominant in older men than postmenopausal women. We speculate that this may be mainly attributable to the abrupt loss of estrogen in these women, besides the gender difference. Specifically, the rapid decrease in estrogen levels after menopause is the most important biological event that causes dramatic changes in homeostasis in women. Regarding muscle metabolism, there is evidence that muscle tissue expresses estrogen receptors and thus estrogen deficiency can directly affect muscle homeostasis [33]. Furthermore, menopause-associated excessive adiposity and the resultant lipotoxicity may also result in negative muscle balance [34]. Therefore, we assume that the effects of these immense changes caused by estrogen deficiency in postmenopausal women may be strong enough to counteract those of chronic low-grade inflammation on muscle metabolism. Further studies focusing on how male and female hormones interact with inflammatory processes in relation to muscle could be highly interesting.

The interaction between bone and muscle has received increasing attention in recent years, because both osteoporosis and sarcopenia, which simultaneously occur in numerous cases, are well-known risk factors for fragility fracture, representing a huge threat to loss of independence in later life [35]. Notably, many researchers have worked hard to identify common contributors explaining the concomitant loss of muscle and bone with aging. In fact, it is now accepted that chronic inflammation is a major cause of osteoporosis [36]. In addition, we here showed that a higher hsCRP level was associated with low muscle strength, a key determinant for sarcopenia, in older general populations. In corroboration with the previous studies related to the pathogenesis of sarcopenia [2,10,18,29,30], our study provides further support that chronic low-grade systemic inflammation could be one of the common risk factors for the concurrent deterioration of muscle and bone with aging. New therapeutic approaches for improving both bone and muscle, based on their highly integrated nature, are necessary for effective fracture prevention.

The major strength of our study is that it is a large population-based study, using well-collected national data, which enhances the statistical reliability of the results and the generalizability of the data. We also used the Korean-specific HGS cut-off point for low muscle strength, as recommended by the Asian Working Group for Sarcopenia [2] and European Working Group on Sarcopenia in Older People [10], because these values can vary, depending on ethnicity, body size, lifestyle, and cultural backgrounds. Despite these strengths, several limitations should be considered when interpreting our data. Most importantly, because it was a cross-sectional study, we could not determine whether there was a causal relationship between dietary protein and the composite indices. Second, the information about muscle mass is not available in the Sixth Korea National Health and Nutrition Examination Survey (KNHANES VI). However, low HGS is known as a clinical marker of poor mobility and a better predictor of clinical outcomes than low muscle mass [10,37] and muscle mass and strength are not linearly related [11,12]. Hence, this study has clinical meaning, in demonstrating the importance of chronic low-grade inflammation in muscle health during aging. Third, HGS and blood sampling were not performed in approximately 12% out of 6,693 participants aged ≥10 years due to inappropriate conditions for tests or their disagreement. Lastly, we cannot exclude the possibility that the observed association could have resulted from uncontrolled factors that affect serum hsCRP level and/or muscle metabolism, such as 25-hydroxyvitamin D level.

In summary, data gathered from a nationally representative cohort illustrate that higher serum hsCRP is associated with a lower HGS and higher risk for low muscle strength in older men. However, these associations are not significant in postmenopausal women. These findings provide the clinical evidence that the detrimental effects of systemic inflammation on muscle, mainly observed in patients with pathologic diseases, could be applied to relatively healthy general populations, and even chronic subclinical low-grade inflammation may contribute to muscle deterioration during aging, especially in men.

## METHODS

### Study population

This cross-sectional study was based on data acquired by the third year (2015) of the KNHANES VI (2013–2015). This nationwide survey used a stratified, multistage, clustered probability sampling method, to select a representative sample of the non-institutionalized, civilian Korean population, as described previously [38,39]. In brief, a three-stage sample design is used for the KNHANES. The primary sample units (PSUs) are selected from a sampling frame of all census blocks or resident registration addresses. Each PSU consists of approximately 50-60 households. Following the selection of PSUs, all dwelling units in the PSU are listed and 20 households are selected through the field survey for household screening. The final stage of selection occurs in the household, where all members aged 1 year or older are selected to participate. Approximately 10,000 persons are sampled in total in all 192 PSUs per year. The expected total sample size is based on past KNHANES waves using the response rates for each subdomain of interest. The goal for the overall response rate for the KNHANES is 75%. The survey provides extensive data on health and nutrition collected by health interview, nutrition interview, and health examination, which is publicly available at the KNHANES website (http://knhanes.cdc.go.kr; available in Korean and English). In 2015, the surveys were completed by 7,380 participants aged 1 year or older. Among them, participants aged ≥10 years were 6,693. HGS was measured in all participants aged ≥10 years with their consents, except for those who were excluded based on the following criteria: no hands, arms, or thumbs/ paralysis of hands/ cast on hands or fingers/ bandage on hands or wrist/ hand or wrist surgery in the prior 3 months/ pain, tingling, or stiffness in hands or wrist within the prior week (*n* = 5,905). Blood samples including serum hsCRP levels were also obtained in all participants aged ≥10 years with their consents, except for those with hemophilia or with rash, open wound, weak blood vessels, vascular occlusion, paralysis, or a shunt for hemodialysis on both arms, and except for those have received anti-coagulant or chemotherapy within 1 month (*n* = 5,860). Consequently, information about both HGS and serum hsCRP level was available for 5,529 participants aged ≥10 years in 2015. Among them, we enrolled 2,549 participants (1,253 men aged ≥50 years and 1,296 postmenopausal women) for the present study, because we focused on the risk marker related to the deterioration of muscle strength seen with aging. Participants with liver cirrhosis, renal failure, neoplastic diseases, stroke sequelae, or myocardial infarction were excluded from this study (*n* = 169), because these may affect muscle health and/or serum hsCRP level. Participants were also excluded if they had serum liver enzyme (aspartate aminotransferase or alanine aminotransferase) activities above 100 IU/L, increased serum creatinine (≥1.6 mg/dL), or abnormal leukocytes (<4.0 or >10.0 × 10^9^/L) (*n* = 264). All of these criteria were imposed so that subjects with a systemic illness would be excluded. The remaining 2,116 participants (1,036 men aged ≥50 years and 1,080 postmenopausal women) were eligible for this study. Because the KNHANES was a weighted survey, 2,116 participants represented 11,921,911 total participants. All participants in the KNHANES survey provided informed consent. The KNHANES was reviewed and approved by the Ethics Committee of the Korea Centers for Disease Control and Prevention (KCDC).

### Measurement of HGS

HGS was measured using a digital grip strength dynamometer (TKK 5401, Takei Scientific Instruments Co., Ltd., Tokyo, Japan), which measures between 5.0 and 100.0 kg of force and has an adjustable grip span. The minimum measurement unit is 0.1 kg. During the assessment, participants were asked to stand upright with their feet hip-width apart and to look forward with the elbow fully extended. The dynamometer was held by the testing hand in a neutral, comfortable position (not flexed or extended) with 90° flexion at the index finger. Participants performed three trials for each hand alternately, always starting with the dominant hand. Participants were instructed to squeeze the grip continuously with full force for at least 3 seconds. The time between each trial was approximately 60 seconds. Grip strength was defined as the maximally measured grip strength of the dominant hand [27,40]. In this study, we adopted the Korean-specific HGS cut-off point for the definition of “low muscle strength”, and the HGS cut-off values in male and female elderly aged ≥65 years were 28.6 and 16.4 kg, respectively, based on the population-based study [27].

### Measurements of clinical and laboratory parameters

All subjects underwent a thorough physical examination. Age, body weight, height, smoking and drinking habits, and resistance exercises, such as push-up, sit-up, dumbbell, and horizontal bar, were recorded. Three levels of smoking (never, past, or current), drinking habits [no, moderate (1–3 times/week), or heavy (≥4 times/week)], and resistance exercise [no, intermittent (1-3 days/week), or regular (≥ 4 days/week)] were identified. Height (cm) and weight (kg) were measured by using standardized protocols while the subject was dressed in light clothing without shoes. BMI (kg/m^2^) was calculated from the height and weight. For women, self-reported questionnaires were used to assess menopausal status. BP was measured on the right arm using a standard mercury sphygmomanometer (Baumanometer Wall Unit 33(0850), W.A. Baum Co. Inc., Copiague, NY, USA), with the participant in the sitting position. Systolic and diastolic BP readings were recorded twice at 5 minute intervals and averaged for analysis. Blood samples were collected from the antecubital vein of each participant in the morning after overnight fasting for at least 8 hours, immediately refrigerated, and then transported to the Central Testing Institute (Neodin Medical, Inc., Seoul, Korea), where they were analyzed within 24 hours after transport. Fasting plasma glucose and serum total cholesterol levels were measured by hexokinase UV and enzymatic method, respectively, using a Hitachi automatic analyzer 7600-210 (Hitachi Ltd, Tokyo, Japan). Serum hsCRP concentration was measured by immunoturbidimetry, using a Cobas bio-centrifugal analyzer (Roche, Germany). The lower detection limit of the kit was serum hsCRP <0.100 mg/L and the coefficient of variation (CV) value was <5%.

### Statistical analysis

Based on the statistical guidelines of the KCDC, all analyses were performed using the Complex Samples Plan (CSPLAN), which is available as the complex samples option in SPSS version 18.0 (SPSS, Inc., Chicago, IL, USA). Survey sample weights were considered in all analyses, to produce estimates that were representative of the non-institutionalized civilian population in Korea [41]. Data are presented as means with 95% confidence intervals (CIs), or as percentages unless otherwise specified. The baseline characteristics were calculated using an unpaired *t-*test for continuous variables or the χ2 test for categorical variables. To test our hypothesis that a higher serum hsCRP level might be associated with a lower HGS, we performed multiple linear regression analyses using an Enter method, with HGS as a dependent variable and serum hsCRP level as an independent variable. Potential confounding factors included age, BMI, smoking and drinking habits, and resistance exercise, fasting plasma glucose, serum total cholesterol, and systolic blood pressure, which were selected based on clinical applicability and/or statistical significance in univariate analyses. The beta coefficient in this model signifies how much the mean of the dependent variable changes given a one-unit shift in the independent variable. To further analyze the HGS changes according to serum hsCRP quartiles, multivariable-adjusted least-square means with 95% CIs were estimated and compared by analysis of covariance (ANCOVA) after adjustment for confounders. The trend of HGS across increasing quartiles of serum hsCRP level was checked by examining *P-*values for the trends, using multiple linear regression analyses. The multivariable-adjusted least-square means with 95% CIs of serum hsCRP regarding the presence of low muscle strength were also estimated and compared by ANCOVA. To generate ORs with 95% CIs for low muscle strength per the SD increment in serum hsCRP, we performed multiple logistic regression analyses after adjustment for confounding variables. *P* <0.05 was considered to indicate statistical significance.

## Supplementary Material

Supplementary Table
