# Supplementary material for: Inverse relationship between serum hsCRP concentration and hand grip strength in older adults: a nationwide population-based study
Source: Aging (Albany NY). 2018 Aug 16;10(8):2051–61. doi: 10.18632/aging.101529 (PMC6128433; doi:10.18632/aging.101529)
Supplement: Supplementary Table [file aging-10-101529-s001.pdf]

Supplementary Table 1. Multiple linear regression analysis to determine whether serum hsCRP level is independently associated with hand grip strength after the stratification according to smoking, drinking, and resistance exercise status in men.

Stratification	Dependent variable: hand grip strength		
	β	SE	*P*
Smoking status			
Never smoking group	–0.190	0.233	0.417
Past smoking group	**–0.225**	**0.104**	**0.032**
Current smoking group	**–0.399**	**0.201**	**0.048**
Alcohol drinking status			
No drinking group	**–0.224**	**0.086**	**0.010**
Moderate drinking group	–0.253	0.165	0.129
Heavy drinking group	–0.583	0.364	0.113
Resistance exercise status			
No exercise group	**–0.290**	**0.105**	**0.007**
Intermittent exercise group	–0.249	0.256	0.334
Regular exercise group	**–0.312**	**0.149**	**0.040**

The Enter method is applied to this model with hand grip strength (kg) as a dependent variable, and with serum hsCRP level (mg/L) as an independent variable. Multivariable adjustment model includes age, body mass index, fasting plasma glucose, serum total cholesterol, and systolic blood pressure as confounding factors. Bold numbers indicate statistically significant values. β, regression coefficient; SE, standard error; hsCRP, high sensitivity C-reactive protein.
